# Robotic salvage partial nephrectomy following surgical and ablative therapies

**DOI:** 10.1590/S1677-5538.IBJU.2024.0117

**Published:** 2024-03-30

**Authors:** Carol L. Feng, Antonio Franco, Francesco Ditonno, Celeste Manfredi, Alexander K. Chow, Riccardo Autorino

**Affiliations:** 1 Rush University Medical Center Department of Urology Chicago IL USA Department of Urology, Rush University Medical Center, Chicago, IL, USA; 2 Sapienza University Sant'Andrea Hospital Department of Urology Rome Italy Department of Urology, Sant'Andrea Hospital, Sapienza University, Rome, Italy; 3 University of Verona Department of Urology Verona Italy Department of Urology, University of Verona, Verona, Italy; 4 "Luigi Vanvitelli" University Urology Unit Naples Italy Urology Unit, "Luigi Vanvitelli" University, Naples, Italy

## Abstract

**Purpose:**

Partial nephrectomies in the salvage setting after ablative or surgical therapy remain challenging cases that are underreported in the literature (1-5). The aim of this video is to demonstrate techniques for robotic salvage partial nephrectomy to manage recurrent renal cell carcinoma (RCC) after failed prior partial nephrectomy and primary cryotherapy.

**Materials and methods:**

A 55-year-old man after previous robotic-assisted right partial nephrectomy presented with a 2.5 cm locally recurrent renal mass abutting the collecting system. A 59-year-old man with right renal cell carcinoma initially treated with cryoablation presented local recurrence. CT imaging demonstrated 2.6 cm right renal mass consistent with tumor recurrence at previous treatment site.

**Results:**

Both procedures were completed in under 180 minutes. Clamp time was 22 minutes after the previous partial nephrectomy and 25 minutes after previous cryotherapy. There were no perioperative complications. Pathology in both cases demonstrated pT1a clear cell RCC with negative margins. Both patients have since no evidence of recurrent disease on follow-up imaging at 1 and 2 years, respectively.

**Conclusions:**

Salvage robotic partial nephrectomy should be considered as a feasible treatment option after failure of initial therapy—surgical or ablative. A salvage procedure is often more challenging than its standard therapy-naïve counterpart due to development of dense inflammation after previous interventions. Despite this, robotic partial nephrectomies in the salvage setting can be safely carried out with good surgical outcomes, particularly when utilizing intraoperative ultrasound to identify tumor margins and key anatomy.

**Available at:** http://www.intbrazjurol.com.br/video-section/20240117_Autorino_et_al

**Int Braz J Urol. 2024; 50 (Video #5): 373-4**
